# 

**Published:** 1853-04

**Authors:** 


*Meeting of the American Medical Association.* — We would remind our readers of the annual meeting of the American Medical Association, on the first Tuesday in May, at the city of New York. The attendance will probably be larger than at any previous meeting owing to the facilities of traveling in every direction to the great American metropolis, and the many attractions which the city of New York present, aside from those pertaining to the association. We do not presume too much (although it is quite unnecessary) in giving assurances of a hospitable reception by our brethren in New York.

---

*Western Lancet.* — Dr. Thomas Wood has lately been associated with Dr. L. M. Lawson in the editorial management of the above journal. The surgical department of the work is assigned to Dr. Wood.

*Death of Dr. Pereira.*— Died at London, Jan. 20th, Jonathan Pereira M. D., aged 49.

The name of Dr. Pereira is familiar to the profession of this country by his most valuable works on food and diet, and the materia medica. Science has lost an able and industrious laborer.

---

*Chrono-thermal Medical School.*— The Philadelphia Med. and Surg. Journal states, that the Legislature of Penneylvania have granted a charter for a medical college denominated *Chrono-thermal*, and that a spring course of lectures is announced. Quackery appears to be particularly rampant in the ancient seat of medical science.

---

*Transactions of the American Medical Association.* — We perceive by our more favored exchanges, that the volume of the Transactions for 1852, has been issued. A copy has not yet reached us. We must offer this as our apology for not offering some notice of its contents.

---

*Transactions of the Kentucky State Medical Society.*— This publication has been received, but too late to be noticed in the present issue. It will receive attention in our next No.

---

Owing to an overplus of Editorial matter prepared for this No., some articles are omitted. Among these are notices of numerous introductory lectures received during the last few months. A letter from a correspondent on *hats and baldness*, is in type and will appear in our next issue. Several new publications have been received which, will be noticed hereafter.

### REPORT ON CHRONIC PLEURISY, BY AUSTIN FLINT, M. D.

THIS Report has been neatly published in a separate form, and may be ordered from the house of DERBY, ORTON & MULLIGAN, Booksellers, Buffalo, N. Y. The price is 50 cents per copy. It can readily be sent by mail.

---

### NOTICE.

A PRACTITIONER in one of the most eligible country situations in Western New York, contemplating a change of residence to the city, will dispose of house and lot, horse, etc., on favorable terms, to a well educated physician desirous of succeeding him in practice.

Communications may be made through the Editor of the Buffalo Medical Journal.

